# The role of thickness on the structural and luminescence properties of Y_2_O_3_:Ho^3+^, Yb^3+^ upconversion films

**DOI:** 10.1038/s41598-024-68367-x

**Published:** 2024-08-01

**Authors:** Vhahangwele Makumbane, Robin E. Kroon, Mubarak Y. A. Yagoub, Lucas J. B. Erasmus, E. Coetsee, Hendrik C. Swart

**Affiliations:** Department of Physics, University of the Free State, Bloemfontein, 9300 South Africa

**Keywords:** Y_2_O_3_:Ho^3+^, Yb^3+^, Upconversion, Pulsed laser deposition, Thin films’ thickness, Materials science, Optics and photonics

## Abstract

The structural, surface, and upconversion (UC) luminescence properties of Y_2_O_3_:Ho^3+^,Yb^3+^ films grown by pulsed laser deposition, for different numbers of laser pulses, were studied. The crystallinity, surface, and UC luminescence properties of the thin films were found to be highly dependent on the number of laser pulses. The X-ray powder diffraction analysis revealed that Y_2_O_3_:Ho^3+^,Yb^3+^ films were formed in a cubic structure phase with an Ia $\overline{3 }$ space group. The thicknesses of the films were estimated by using cross-sectional scanning electron microscopy, depth profiles using X-ray photoelectron spectroscopy (XPS), and the Swanepoel method. The high-resolution XPS was used to determine the chemical composition and oxidation states of the prepared films. The UC emissions were observed at 538, 550, 666, and 756 nm, assigned to the ^5^F_4_ → ^5^I_8_, ^5^S_2_ → ^5^I_8_, ^5^F_5_ → ^5^I_8_, and ^5^S_2_ → ^5^I_7_ transitions of the Ho^3+^ ions. The power dependence measurements confirmed the involvement of a two-photon process in the UC process. The color purity estimated from the Commission International de I’Eclairage coordinates confirmed strong green UC emission. The results suggested that the Y_2_O_3_:Ho^3+^,Yb^3+^ UC transparent films are good candidates for various applications, including solar cell applications.

## Introduction

Transmission losses, which describe the incident energy lost to unabsorbed low-energy photons, are one of the significant loss processes in solar cells^1^. Upconversion (UC) can convert the low-energy photons that are unabsorbed by solar cells into high-energy photons. These high-energy photons can, therefore, be absorbed by the solar cell. This process can reduce transmission losses, and in turn, boosts the efficiency of the solar cells^2^. Thus far, materials reported that display visible UC emissions focused more on ceramics or powders^3–11^, which limits their practical solar cell use due to their poor compatibility with solar cell technology. Therefore, UC transparent films are more suitable for applications due to their good compatibility with solar cell technology. For solar cell applications, UC materials are applied underneath the solar cells as a layer with a controllable thickness, therefore, this film study is more suitable for such applications^12^.

As a typical host for a variety of luminescent materials, Y_2_O_3_ exhibits wide transmittance from 0.2 to 8 μm^12^, which is wide enough to cover the whole solar spectrum for solar cells. Furthermore, Y_2_O_3_ is an important host material for UC due to its excellent chemical durability, thermal stability, wide bandgap of 5.8 eV, low phonon energy of ~ 500 cm^−1^, and high refractive index of ~ 2^4,13,14^. Doping Y_2_O_3_ with Ho^3+^ ions can offer certain advantages for UC since Ho^3+^ ions have been studied as excellent UC activators due to their high UC efficiency^15^. However, to enhance the UC efficiency further, the Yb^3+^ ion is typically co-doped as an ideal UC sensitizer for the Ho^3+^ ion due to its large absorption cross-section in the near-infrared (NIR) region around 980 nm^16^. Y_2_O_3_ thin films doped with lanthanide ions have been the subject of numerous studies^17–22^. Qiao et al.^17^ reported the UC properties of Y_2_O_3_:Er films prepared by the sol–gel method and obtained red and green emissions. Dikovsa et al.^18^ prepared the Er, Yb co-doped Y_2_O_3_ thin films by pulsed laser deposition (PLD) and studied the structural and optical properties of the prepared films. The authors obtained a smoother surface and a better crystalline structure for the Er, Yb co-doped Y_2_O_3_ films deposited at low oxygen pressure. Lian et al.^19^ reported on the deposition of the Eu^3+^ doped Y_2_O_3_ ultrathin films deposited on alumina nanoparticles by a solution synthesis method. The authors observed a strong photoluminescence (PL) in the film annealed at high temperatures. Pandey et al.^23^ investigated the temperature-induced UC behavior in Ho^3+^,Yb^3+^ co-doped Y_2_O_3_ thin films using PLD and obtained an intense green polycrystalline film of high quality with a smooth surface. They barely addressed the thickness effect on the UC emission of the prepared films, except for the thickness obtained from the Auger results. However, considering UC films as coatings on solar cells, the thickness of the film is vital. Hence, investigating the role of the Y_2_O_3_:Ho^3+^, Yb^3+^ UC thin film thickness is essential.

In this work, Y_2_O_3_:Ho^3+^, Yb^3+^ UC thin films were prepared using the pulsed laser deposition (PLD) technique for a different number of laser pulses. The structural, surface, and optical properties of the PLD Y_2_O_3_:Ho^3+^, Yb^3+^ UC films were investigated using X-ray powder diffraction (XRPD), X-ray photoelectron spectroscopy (XPS), scanning electron microscopy (SEM), and atomic force microscopy (AFM). The UC luminescence emissions were produced by exciting the thin films under 980 nm laser excitation. The photons involved in the UC process were confirmed by power dependence measurements.

## Experimental

The Y_2-x-y_O_3_:Ho_x=0.005_,Yb_y=0.05_ films were prepared by the PLD method using the Nd:YAG laser with a wavelength of 266 nm under oxygen (O_2_) gas pressure of 50 mTorr and a various number of laser pulses (10,000, 20,000, 30,000, 40,000, 50,000, and 60,000). The PLD target was prepared by mixing 8 g of Y_2-x-y_O_3_:Ho_x=0.005_, Yb_y=0.05_ powder with ethanol (99.9%) and compressed at 75 tons for an hour to produce a pellet (target). Ethanol was used as a binder to help compact the powder more evenly. The target was then annealed at 1110 °C for 10 h to ensure that all impurities and ethanol had been eliminated. The preparation procedure of the Y_2-x-y_O_3_:Ho_x_,Yb_y_ powders was previously reported by Makumbane et al.^24^. The researchers studied the UC luminescence of Y_2-x-y_O_3_:Ho_x=0.005_,Yb_y_ (y = 0, 0.002, 0.006, 0.01, 0.05, 0.1, 0.2) phosphors. The UC emission intensity was optimized at a concentration of y = 0.05 Yb_3+_. Hence, Y_2-x-y_O_3_:Ho_x=0.005_,Yb_y_ = 0.05 was used to prepare a pellet for this study. Moreover, the difference in the optimal concentrations with the work done by Pandey et al.^23^ could be due to the differences in the synthesis conditions used. They have used different starting materials, and annealing temperatures to synthesized their phosphor materials.

The deposition was carried out on soda lime glass substrates. The substrates were thoroughly cleaned for 15 min in an ultrasonic bath using acetone, ethanol, and distilled water. Nitrogen gas was then used to dry the cleaned substrates. The prepared target and a single cleaned substrate were then placed inside the PLD chamber system, where the chamber was pumped down to a base pressure of 1.5 × 10^−5^ Torr and back-filled with O_2_ gas at a pressure of 50 mTorr. The target-to-substrate distance was kept constant at 4 cm, while the substrate temperature was kept fixed at 350 °C. The laser energy and repetition rate were approximately 39 mJ/pulse and 30 Hz, respectively.

The structural properties of the Y_2-x-y_O_3_:Ho_x=0.005_,Yb_y=0.05_ films were characterized by XRPD (Bruker AXS GmbH, Karlsruhe, Germany) using a Bruker D8 Advance diffractometer. The morphology and elemental analysis were measured using a JEOL JSM-7800F field emission scanning electron microscope (FE-SEM) equipped with energy-dispersive X-ray spectroscopy (EDS) (JEOL, Tokyo, Japan). Shimadzu SPM-9600 AFM images taken in contact mode were used to determine the surface roughness. The Shimadzu SPM-9600 AFM software was used to examine the topography scans of the surfaces and estimate the root mean square (RMS) roughness. The transmittance was recorded using a Lambda 950 UV–Vis-NIR spectrophotometer (PerkinElmer Ltd., Beaconsfield, United Kingdom). A PHI Quantes Scanning Dual X-ray Photoelectron Microprobe System was used to carry out high-resolution XPS. The XPS spectra fittings were done using Multipack software version 8.2. The depth profile XPS measurements were acquired by bombarding the films with an Ar^+^ ion sputtering gun (2 kV, 1 μA). The sputter rastering area was 1 × 1 mm at a sputter rate of about 15 nm/min. The UC emission measurements were acquired using a 980 nm emitting diode laser as the excitation source. The decay curves were recorded using an FLS980 fluorescence spectrometer (Edinburgh Instruments) equipped with a photomultiplier (PMT) detector with a 980 nm emitting diode laser as the excitation source.

## Results and discussion

### Structural analysis

**Figure 1:**
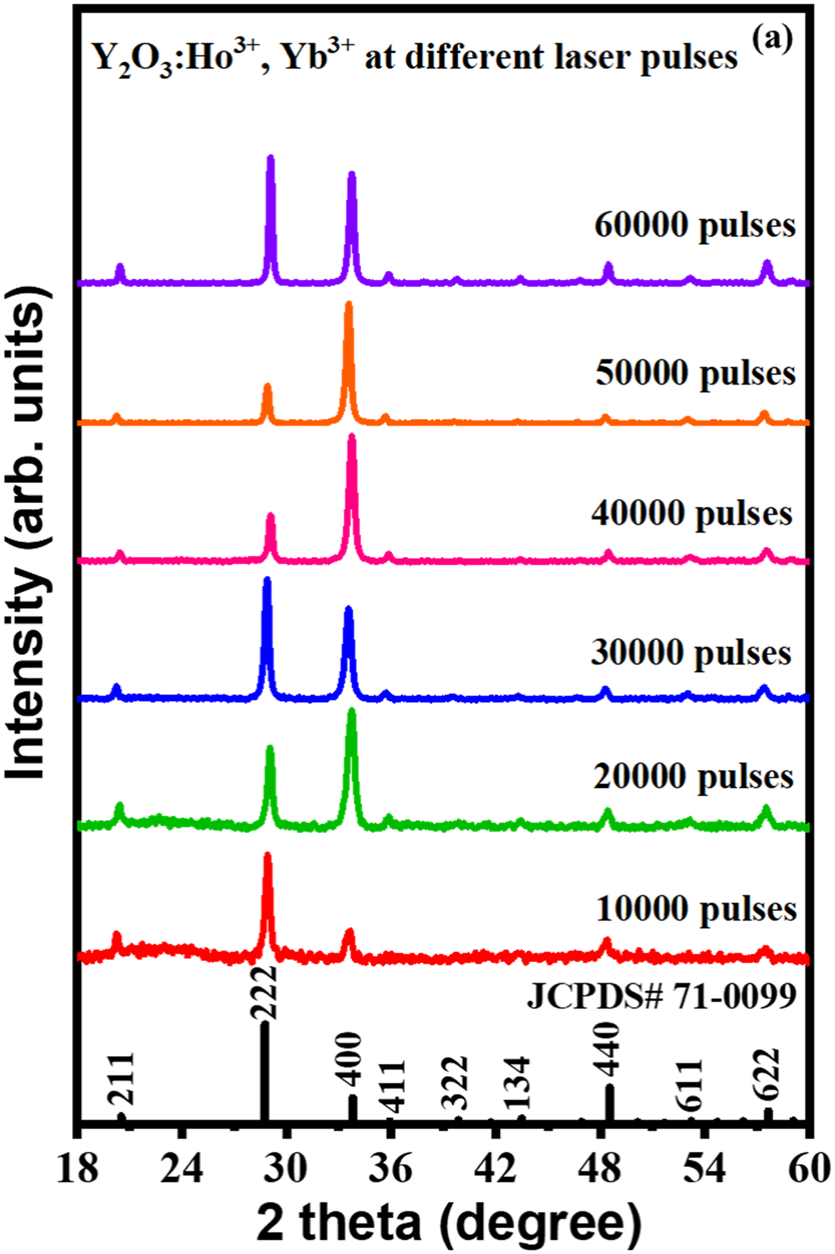

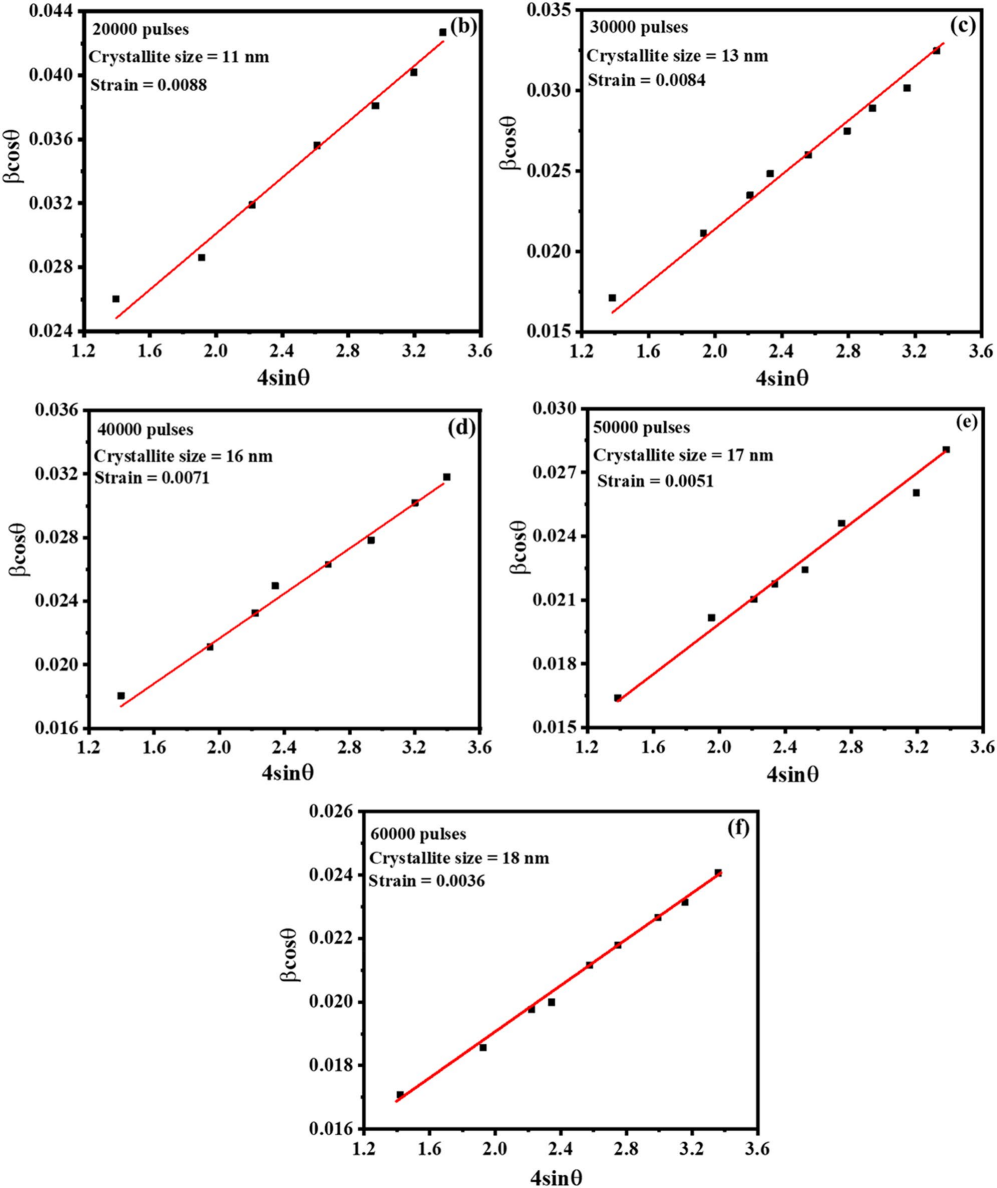
(**a**) XRPD patterns of the Y_2-x-y_O_3_:Ho_x=0.005_,Yb_y=0.05_ thin films deposited at a different number of laser pulses. (**b**–**f**) Williamson–Hall plots of the prepared films deposited at a different number of laser pulses.

Figure 1a displays the XRPD patterns of Y_2-x-y_O_3_:Ho_x=0.005_,Yb_y=0.05_ thin films deposited at different numbers of laser pulses. The patterns of all prepared films are in good agreement with the standard reflection peaks of JCPDS# 71-0099^25^, which indicates that a single-phase cubic structure with an Ia $\overline{3 }$ space group of the Y_2_O_3_ crystal was formed. The films preferred both the (222) and (400) plane orientations, depending on the number of laser pulses. At a low number of laser pulses, the Y_2-x-y_O_3_:Ho_x=0.005_,Yb_y=0.05_ films preferred the (222) plane. With increasing the number of laser pulses, the thin films prefer the (400) plane orientation. This behaviour could be attributed to the oxygen gas pressure of 50 mTorr, which can affect the preferential orientation of the films^26^. Moreover, with increasing the number of laser pulses, some of the defined peaks were observed, which indicates the formation of thicker films and an improvement in the film crystallinity. Structural parameters such as crystallite size (D) and strain ($\varepsilon )$ were estimated using the Williamson-Hall (W–H) equation^27^:

1 $$\beta \text{cos}\theta =4\varepsilon \text{sin}\theta +\frac{k\lambda }{D}$$

where $\lambda$ is the wavelength of radiation (0.154 nm), *k* is a constant related to the crystallite shape (taken as 0.9), $\beta$ is the full width at half-maximum of the diffraction peak, and $\theta$ is the peak position. The lattice strain and average crystallite sizes were estimated by plotting *β*cos*θ* as a function of 4sin*θ* as shown in Fig. 1b–f. The stress of the prepared films was estimated by Hooke’s law which defines the relation between strain and stress within the elastic region ($stress=\sigma \times$ $\varepsilon )$, where the elastic modulus ($\sigma$) of Y_2_O_3_ thin film is 150 GPa^28^.

The average crystallite sizes, lattice strain, and stress are listed in Table 1. The average crystallite size varied between 11–18 nm. It is observed that the crystallite size increased with increasing the number of laser pulses. The increase in the crystallite size is ascribed to the increase in material on the films, which in turn improves the crystallinity of the films. However, the strength of the film, cracking, and hardness have been reported as some of the factors that affect the film’s strain^29^. From Table 1, it can be seen that as the number of laser pulses increased, the strain decreased throughout the lattice, implying that at the lower number of laser pulses, the thin films have a relatively higher lattice mismatch^30^. Additionally, the reduction in strain with an increasing number of laser pulses also indicates the production of thin films with good crystalline properties and a very low number of lattice defects^31^. Liu and Kumar et al.^32,33^ reported that the stress on the thin films can be related to impurities and defects in the crystal (intrinsic stress), the lattice mismatch, growth condition, and mismatch in the thermal expansion coefficient of the thin film, and the substrate (extrinsic stress). As the number of laser pulses increased, the stress gradually decreased, which suggests that increasing the number of laser pulses helps to reduce the tension in the films. It was reported that as the film thickness increases, the lattice disorder decreases because the atoms in the lattice have more time to alter their positions. As a result, the stress tends to be relaxed in thicker thin films^34^.

**Table 1:** Structural parameters of the thin films at different number of laser pulses.

Laser pulses	Crystallite size (nm)	Strain	Stress (GPa)
10,000	–	–	–
20,000	11	0.0088	1.32
30,000	13	0.0084	1.26
40,000	16	0.0071	1.06
50,000	17	0.0051	0.77
60,000	18	0.0036	0.54

### Surface and elemental analysis

**Figure 2:**
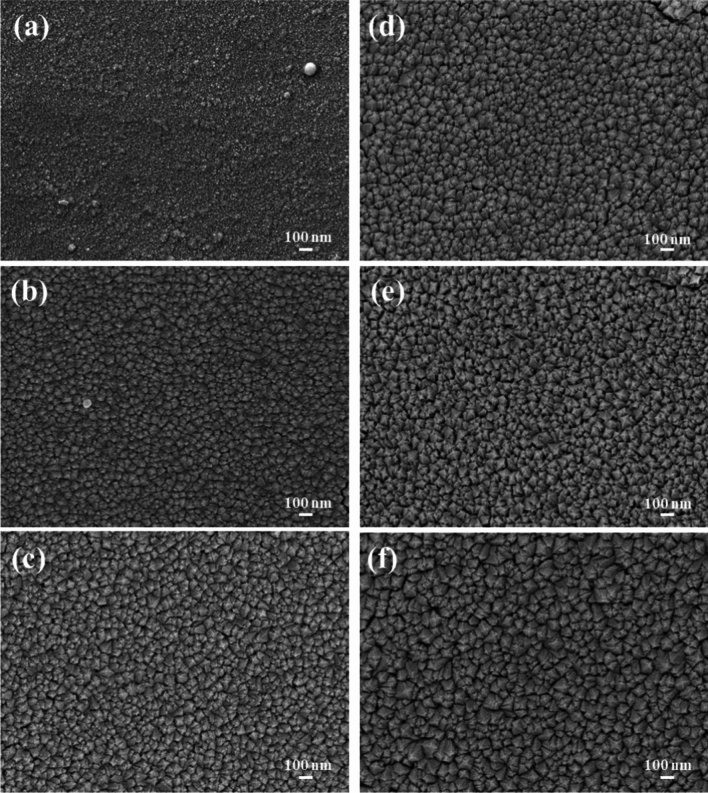
Surface SEM images of the thin films deposited at (**a**) 10,000, (**b**) 20,000, (**c**) 30,000, (**d**) 40,000, (**e**) 50,000, and (**f**) 60,000 pulses.

**Figure 3:**
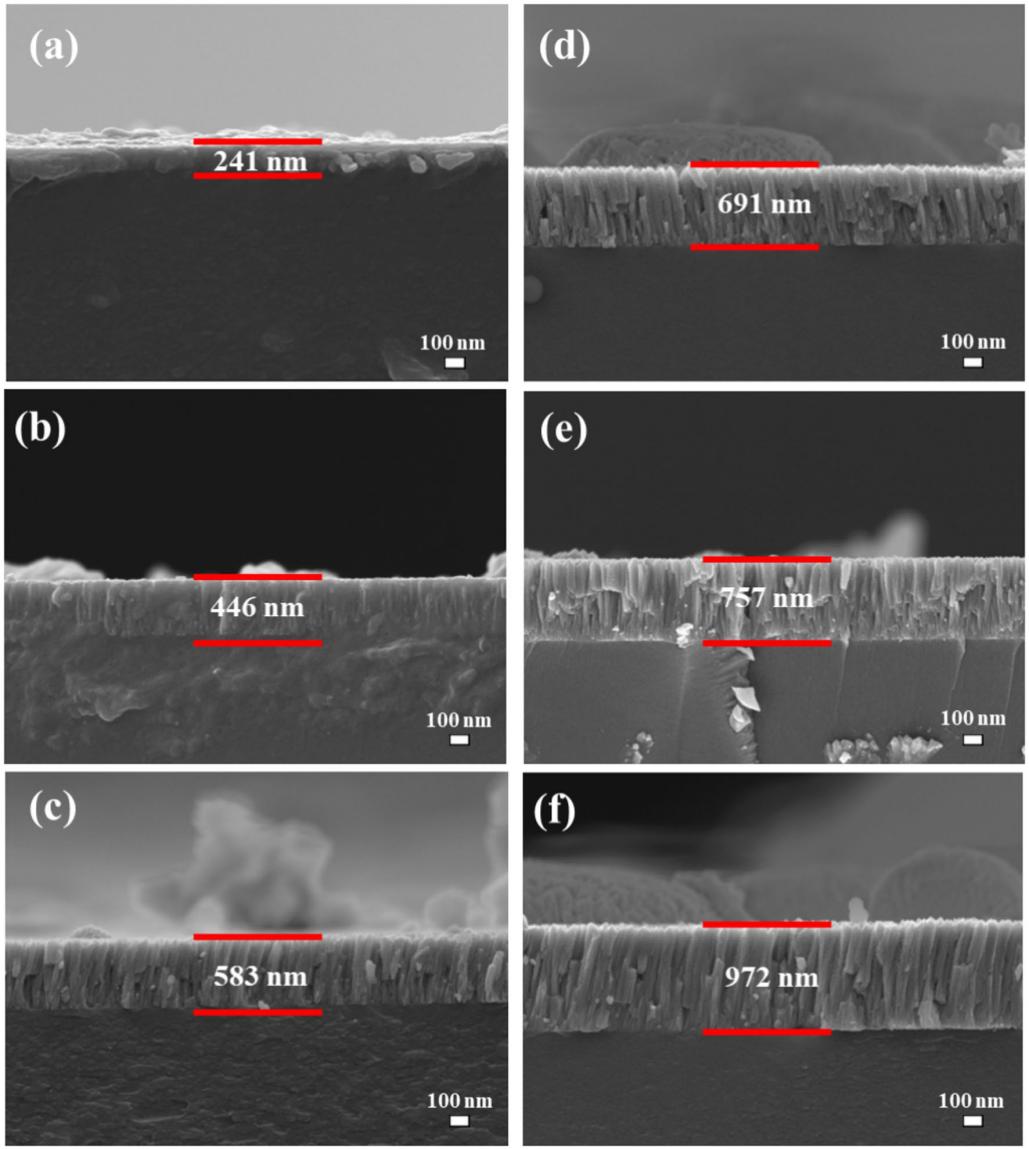
Cross-sectional SEM images of the thin films deposited at (**a**) 10,000, (**b**) 20,000, (**c**) 30,000, (**d**) 40,000, (**e**) 50,000, and (**f**) 60,000 pulses.

Surface and cross-sectional SEM images of the Y_2-x-y_O_3_:Ho_x=0.005_,Yb_y=0.05_ thin films for different number of laser pulses are displayed in Fig. 2. The surface morphology of the films differs depending on the number of laser pulses. When the number of laser pulses increased, the surface morphology of the films exhibited agglomerated, uniform particles. The particle agglomeration occurred during the PLD deposition process, where bigger particles were created when the produced particles started merging^35^. Similar results have been reported by Qu et al.^26^ while studying the highly efficient antireflective downconversion of Y_2_O_3_ films grown by PLD. Furthermore, SEM cross-sectional views were used to determine the thicknesses of the films, see Fig. 3. The thicknesses of the films were determined to be 241, 446, 583, 691, 757, and 972 nm for 10,000, 20,000, 30,000, 40,000, 50,000, and 60,000 pulses, respectively. The thickness of the films increased with the number of laser pulses since more material was deposited on the substrate.

**Figure 4:**
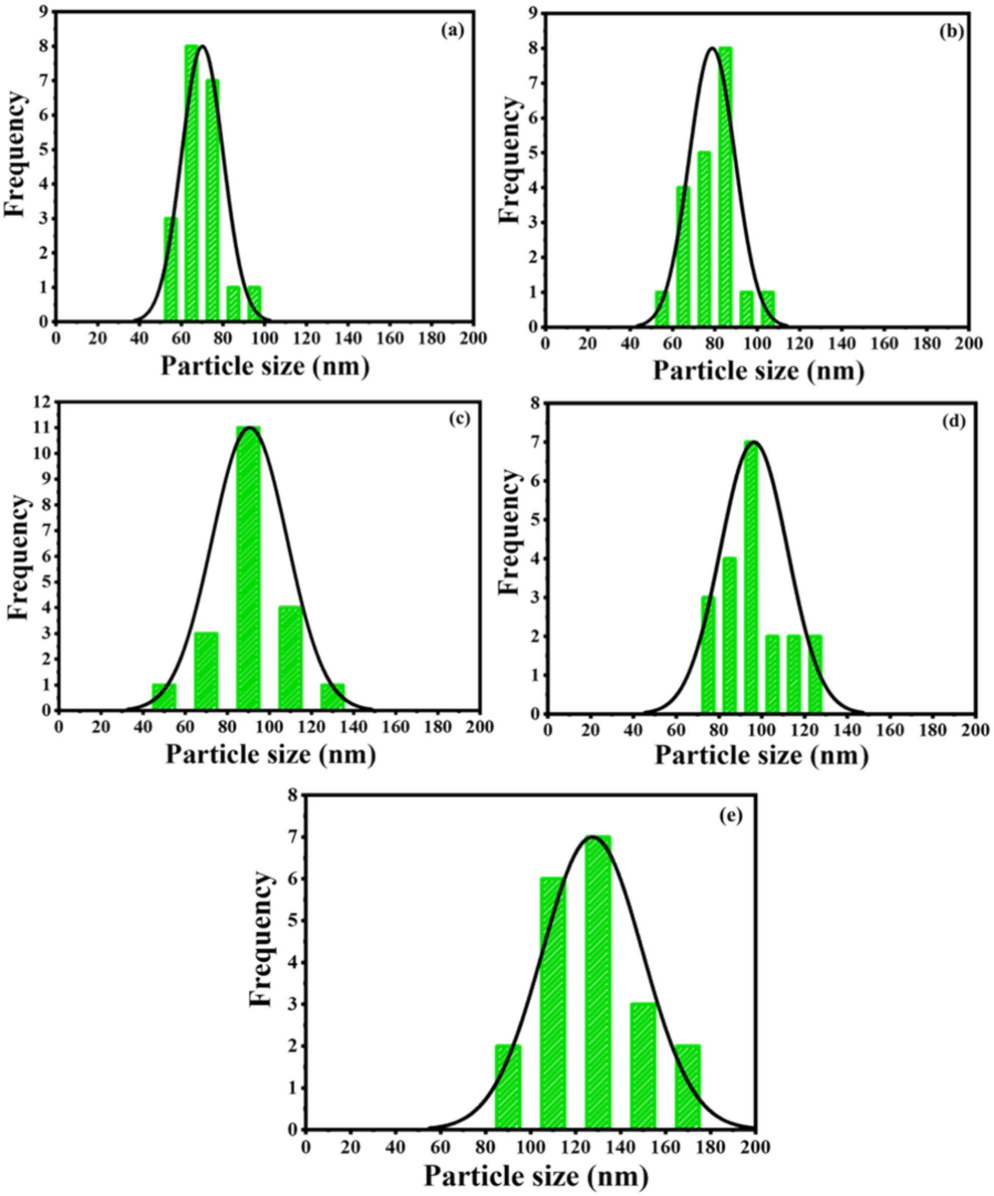
The particle distribution of the prepared films deposited at (**a**) 20,000, (**b**) 30,000, (**c**) 40,000, (**d**) 50,000, and (**e**) 60,000 pulses.

The average particle sizes of the prepared films were calculated using the ImageJ software from surface SEM images. Figure 4a–e displays the histograms of the particle size distribution for the films deposited at images 20,000–60,000 laser pulses. The average particle sizes were found to be 70, 79, 91, 97, and 128 nm for 10,000, 20,000, 30,000, 40,000, 50,000, and 60,000 pulses, respectively. As seen, the average particle size increased due to particle agglomeration at high laser pulses.

**Figure 5:**
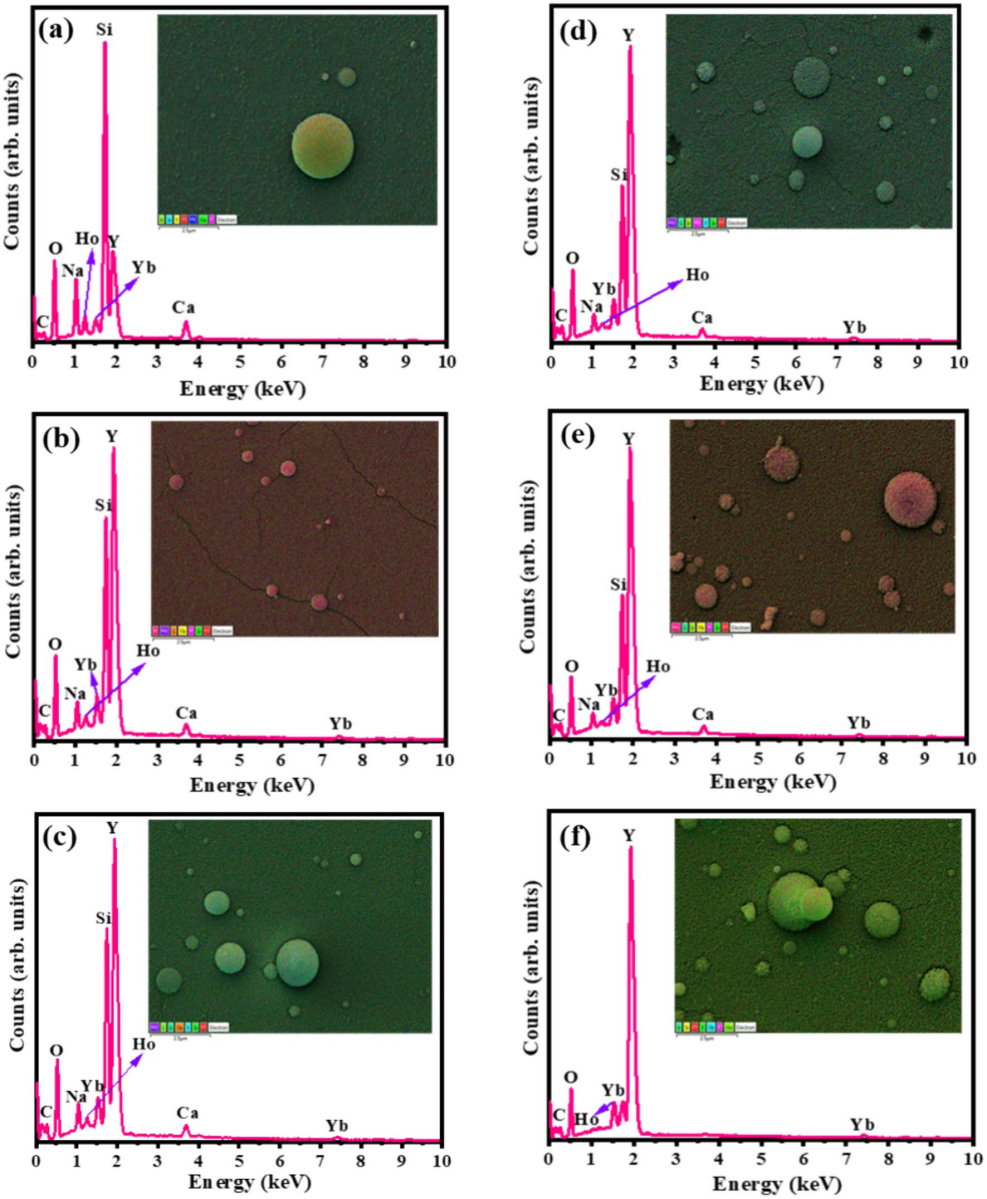
EDS spectra and their corresponding elemental maps (inset) of the films at (**a**) 10,000, (**b**) 20,000, (**c**) 3000, (**d**) 40,000, (**e**) 50,000, and (**f**) 60,000 pulses.

The elemental composition and maps of the Y_2-x-y_O_3_:Ho_x=0.005_,Yb_y=0.05_ films were obtained through the EDS analysis, Fig. 5. All the expected elements (Y, O, Yb, and Ho) in the films were observed. Common impurities such as carbon (C) also appeared in the spectra, which probably could be from the carbon tape used during the measurements. Additionally, the Si, Na, and Ca peaks were detected, which are associated with the chemical composition of the soda-lime glass substrate^36^. As can be seen, the Si, Na, and Ca peaks decreased at higher laser pulses as expected since more material was being deposited on the surface and thicker films were formed. From the elemental maps (inset), all elements were evenly distributed on the surface of the thin films.

**Figure 6:**
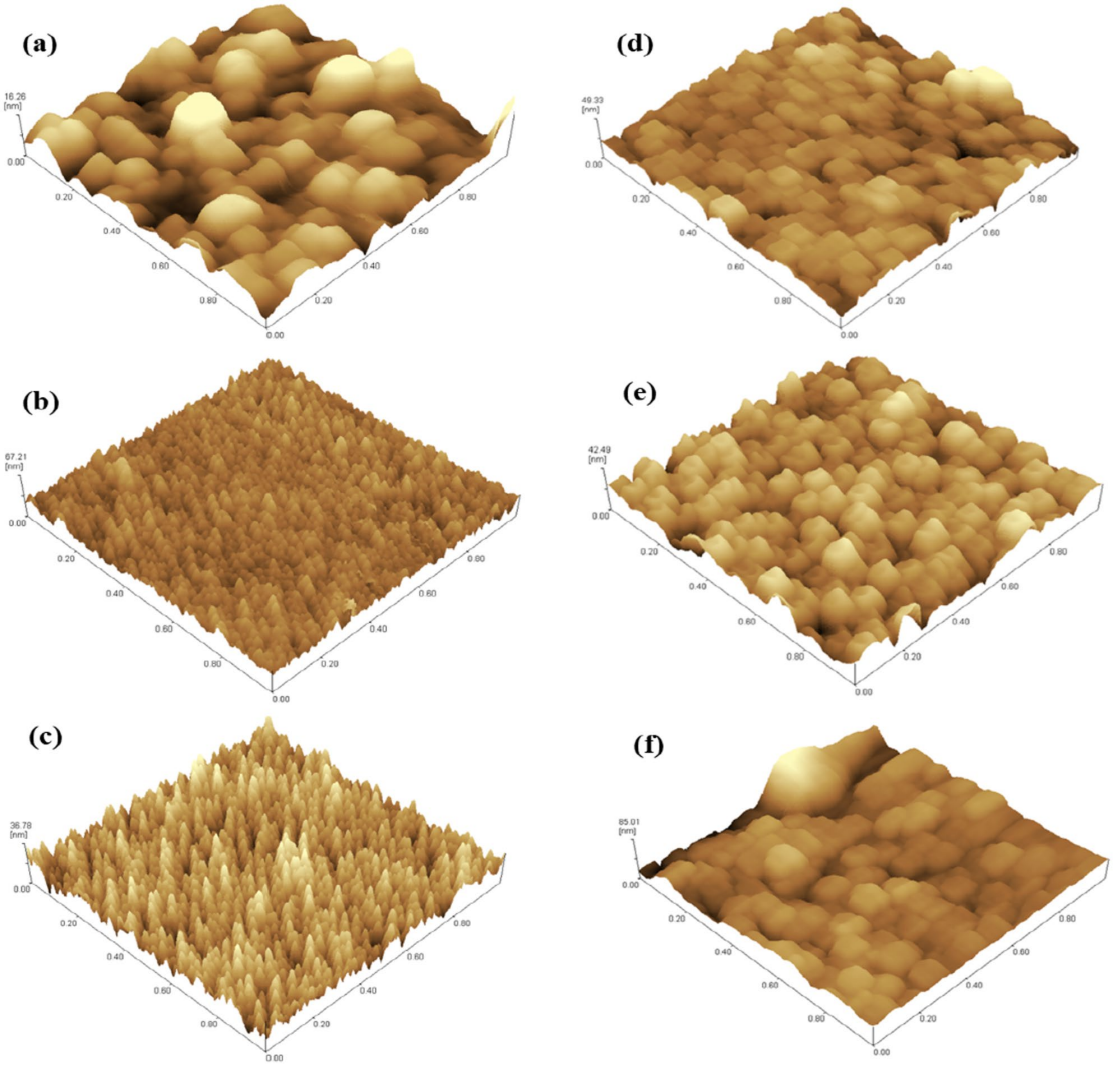
3D AFM images of the thin films at (**a**) 10,000, (**b**) 20,000, (**c**) 30,000, (**d**) 40,000, (**e**) 50,000, and (**f**) 60,000 pulses.

The 3D surface topographic images of the films for different number of laser pulses are presented in Fig. 6. All the AFM images revealed that the films’ surfaces consisted of particles of varying sizes and shapes. The prepared films showed uniformly distributed particles at a higher number of laser pulses. The root mean square (RMS) roughnesses of the thin films grown at laser pulses of 10,000–60,000 were determined to be 3.4, 5.3, 5.7, 6.2, 6.2, and 10.5 nm, respectively. With an increase in laser pulses, the surface roughness of the thin films increased. Peng et al.^37^ studied the effect of thickness on the structural and optical properties of CuI films, where an increase in surface roughness was observed as the thickness of the films increased. Similar observations were reported by Vankhade et al.^38^. The authors investigated the role of thickness on the structural and optical properties of PbS films and reported that as the film thickness increases, the average surface roughness gradually increases. The increase in surface roughness in the films would be expected to enhance the UC emission.

**Figure 7:**
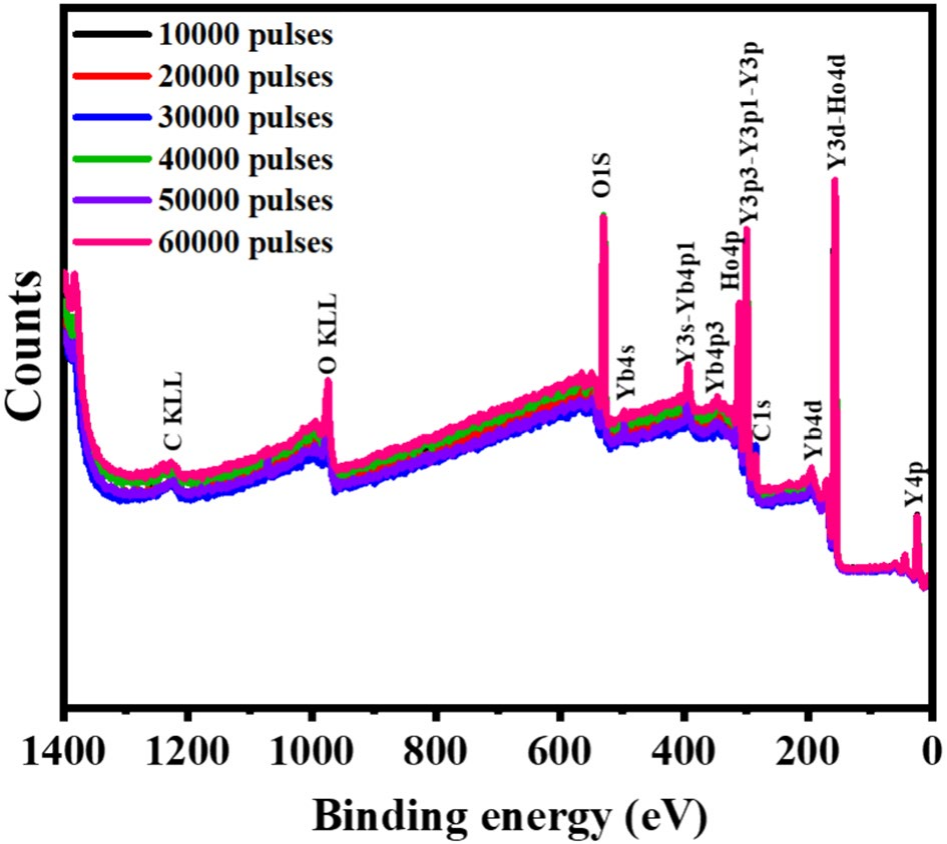
XPS survey of the Y_2-x-y_O_3_:Ho_x=0.005_,Yb_y=0.05_ thin films for different number of laser pulses.

To understand the surface chemical composition of the film, XPS analysis was carried out as displayed in Fig. 7. The XPS survey of the Y_2-x-y_O_3_:Ho_x=0.005_,Yb_y=0.05_ thin films confirmed the presence of Y, O, Ho, and Yb basic elements in the film. The photoelectron peaks of Y 3d, O 1s, Ho 4d, Yb 4d, and C 1s appear at approximately 157.1, 529.2, 157.1, 192.2, and 284.8 eV, respectively^39,40^. The Ho 4d peak normally overlaps with Y 3d, whereas the Yb 4d peak falls under the same energy range as the energy loss peak of Y 3d.

**Figure 8:**
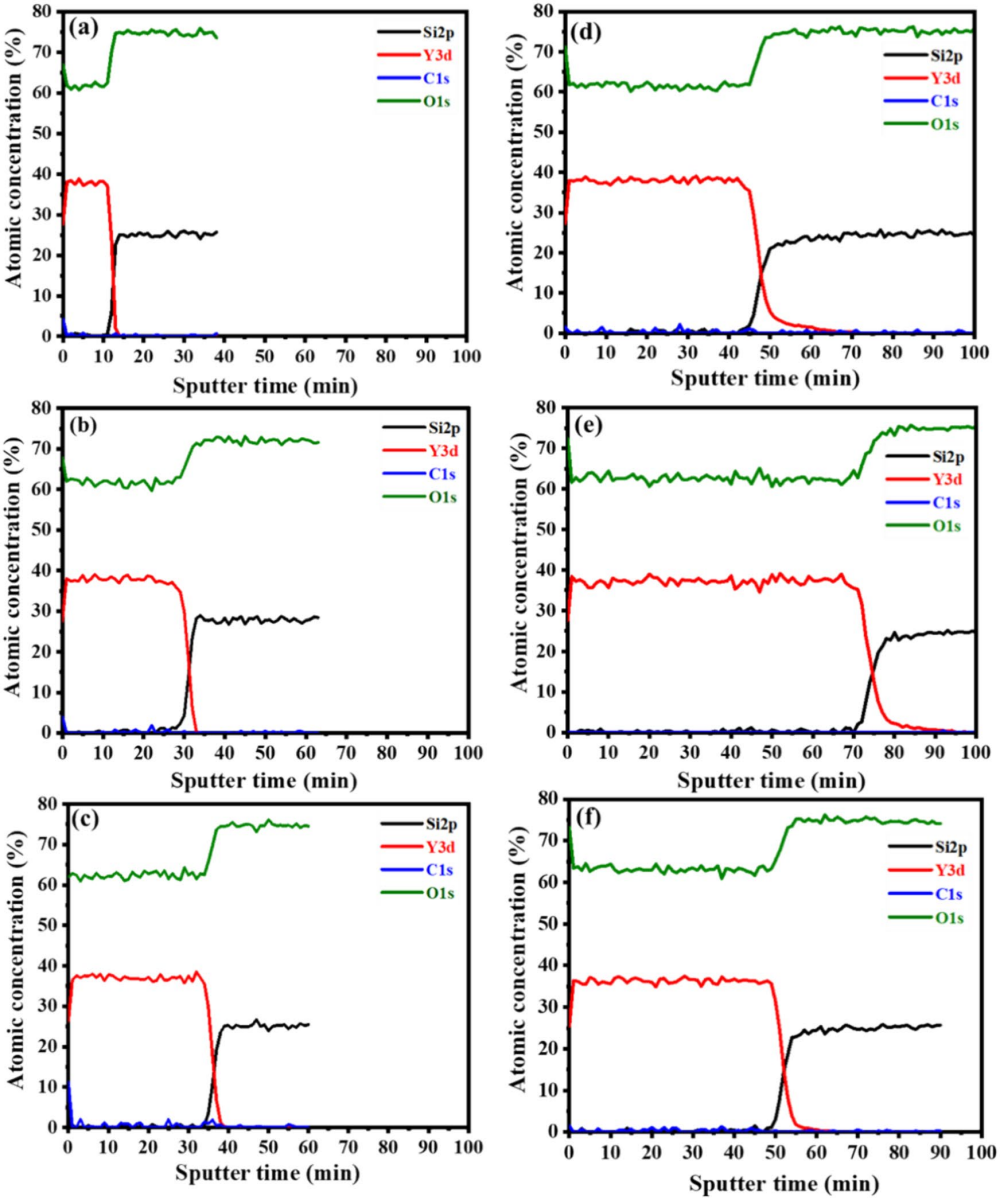
XPS depth profiles of the films at (**a**) 10,000, (**b**) 20,000, (**c**) 30,000, (**d**) 40,000, (**e**) 50,000, and (**f**) 60,000 pulses.

The depth profiling analysis of the thin films for an increasing number of laser pulses is displayed in Fig. 8. All the Y and O main elements have been detected alongside C and Si. The C signal disappeared within a few seconds after sputtering was started, which confirmed that the C was due to surface contamination^41^. From Fig. 8, no segregation of the substrate atoms’ compositions was observed. The soda-lime glass substrates interfaces were reached after 12, 31, 36, 47, 71, and 52 min for the films grown at 10,000, 20,000, 30,000, 40,000, 50,000, and 60,000 laser pulses, which indicates that the films became thicker as the number of laser pulses increased. The observed enhancement in the film thickness is in correlation with the cross-sectional SEM and Swanepoel method results, except for the sample deposited at 50,000 laser pulses, see Table 2. The depth profile for the 50,000 pulses film might have been measured at a position where there were agglomerated particles sitting on the surface of the film, as shown in Fig. 3.

**Table 2:** Comparison between Y_2-x-y_O_3_:Ho_x=0.005_,Yb_y=0.05_ films’ thickness values obtained by cross-sectional SEM, XPS depth profile, and Swanepoel method.

Number of laser pulses	Thickness (nm)		
	Cross-sectional SEM	XPS depth profile	Swanepoel method
10,000	241	180	200
20,000	446	465	267
30,000	583	540	442
40,000	691	705	682
50,000	757	1065	952
60,000	972	780	1218

**Figure 9:**
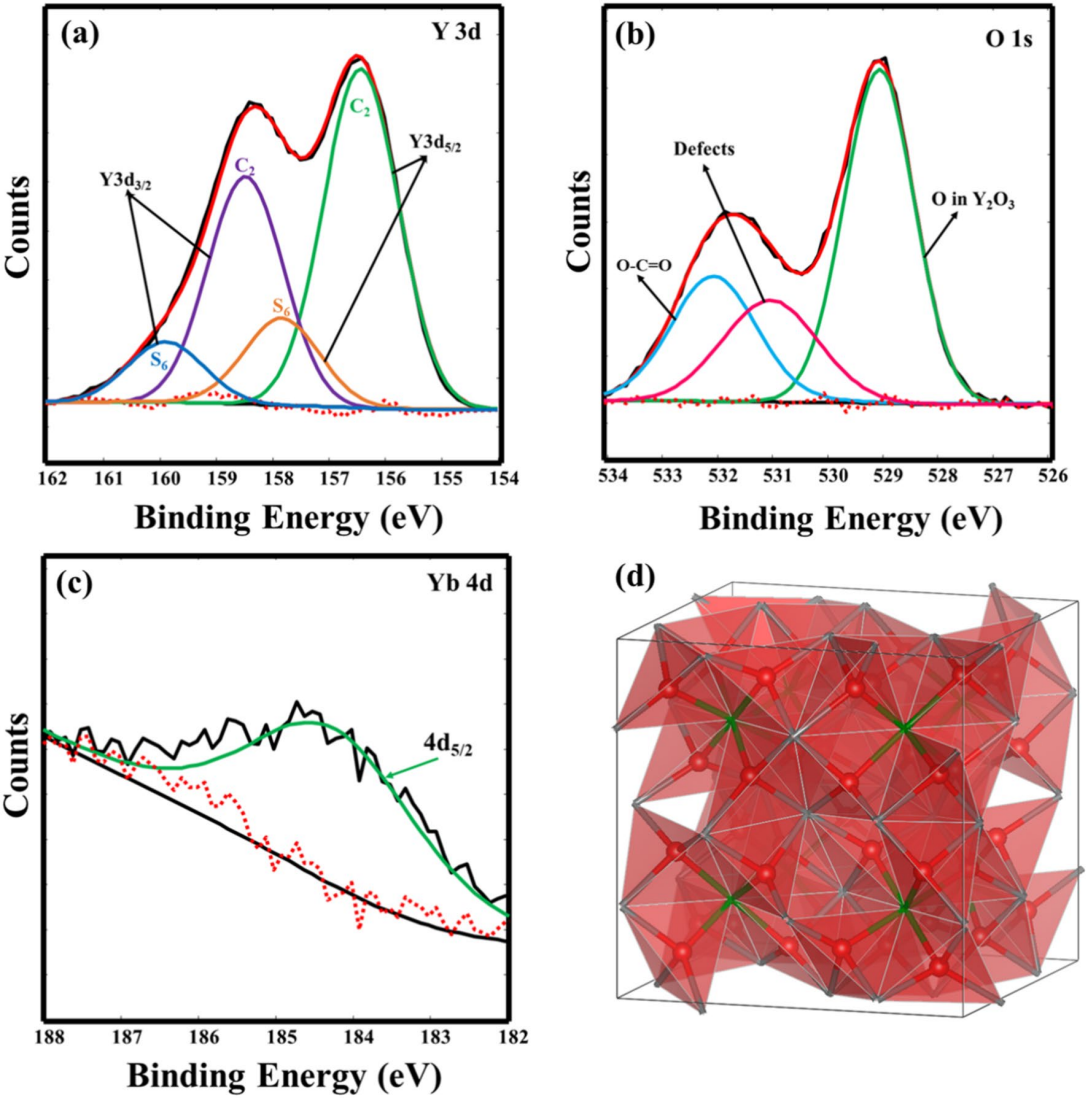
High-resolution XPS core-level spectra for the (**a**) Y 3d, (**b**) O 1s, (**c**) Yb 4d in the Y_2-x-y_O_3_:Ho_x=0.005_,Yb_y=0.05_ films at 60,000 laser pulses, and (**d**) Y_2_O_3_ crystal structure showing O coordination in equivalent positions (Y ions are not shown, but their positions can be inferred from the green and grey bonds).

High-resolution XPS spectra for Y 3d and O 1s peaks are displayed in Fig. 9. In the fitting procedures, the C 1s XPS peak at 284.8 eV was used as a reference. Figure 9a Y 3d spectra fitted into four peaks at 156.4, 157.8, 158.5, and 159.9 eV, which are associated with Y 3d_5/2_ and Y 3d_3/2_ for two different sites (C_2_ and S_6_) of Y^3+^ ion in the Y_2_O_3_ material. The 156.4 and 158.5 eV peaks were the spin–orbit splitting of Y 3d_5/2_ and Y 3d_3/2_ in the C_2_ site, while 157.8 and 159.9 eV peaks were the spin–orbit splitting in the S_6_ site of cubic Y_2_O_3_ crystal, respectively^40,42^. The binding energy separation between the Y 3d_5/2_ and Y 3d_3/2_ peaks was 2.1 eV and the integrated area ratio was 0.7 due to spin-orbital splitting. The Y 3d_5/2_ and Y 3d_3/2_ peak values correlated well with previously reported values in the Y_2_O_3_ structure^43^. The Ho 4d peak overlaps with Y 3d and because the Ho^3+^ concentration in this study was 0.5 mol% there was a low probability of detecting the Ho 4d peak. The high-resolution XPS for the O 1s peak was deconvoluted into three peaks as shown in Fig. 9b. The O 1s peak located at 529.1 eV corresponded to the lattice O in the Y_2_O_3_ host. The peak situated at 531 eV is associated with the O defects inside the material. The binding energy peak positioned at 532.1 eV is ascribed to the O–C=O bond, which might be due to the residual carbon from the fuel (CH_4_N_2_O) used while preparing the target or due to exposure to air^44,45^. Jafer and Shivaramu et al.^43,44^ fitted two peaks of the lattice O corresponding to two different sites in the Y_2_O_3_ host. However, the cubic crystal structure of Y_2_O_3_ (Fig. 9d), shows that all the O are in an identical environment (situated at 48e Wyckoff positions). Each of the O is bonded to 4Y ions (3Y ions at the C_2_ site and 1Y ion at the S_6_ site). The O-Y bond lengths in the C_2_ site are 2.23, 2.24, and 2.27 Å. In the S_6_ site, all the O-Y bond lengths are 2.28 Å^46^. Therefore, the binding energy of the O 1s electron of all these O in the lattice should be the same, thus, a single peak related to the O lattice should be fitted. Moreover, the Yb 4d_5/2_ peak located at 184.7 eV is displayed in Fig. 9c, correlating well with the reported results^47^.

### Optical analysis

**Figure 10:**
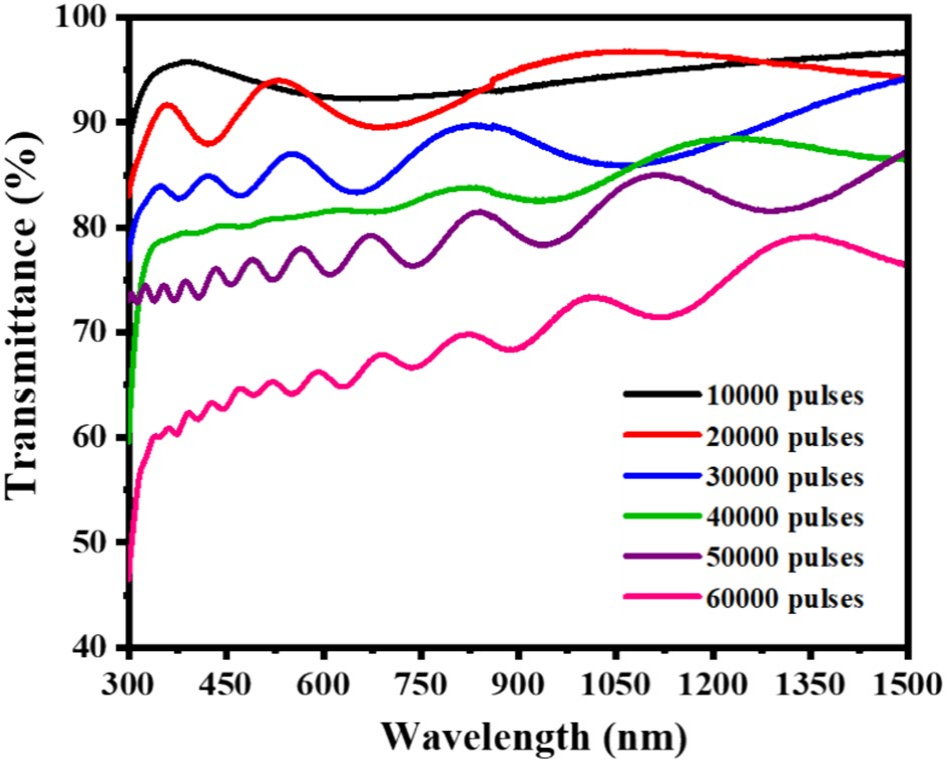
Transmittance spectra of the Y_2-x-y_O_3_:Ho_x=0.005_,Yb_y=0.05_ films at a different number of laser pulses.

The optical transmittance spectra of the Y_2-x-y_O_3_:Ho_x=0.005_,Yb_y=0.05_ films are displayed in Fig. 10, in the 300–1500 nm spectral range. The prepared films exhibited interference fringes which are caused by reflections of the light at the front and back of the film. The interference fringe information implies that the deposited films are uniform and homogeneous^48,49^. It can be seen that the transmittance of the films decreased as the laser pulses increased due to the film thickness effect^50^.

**Figure 11:**
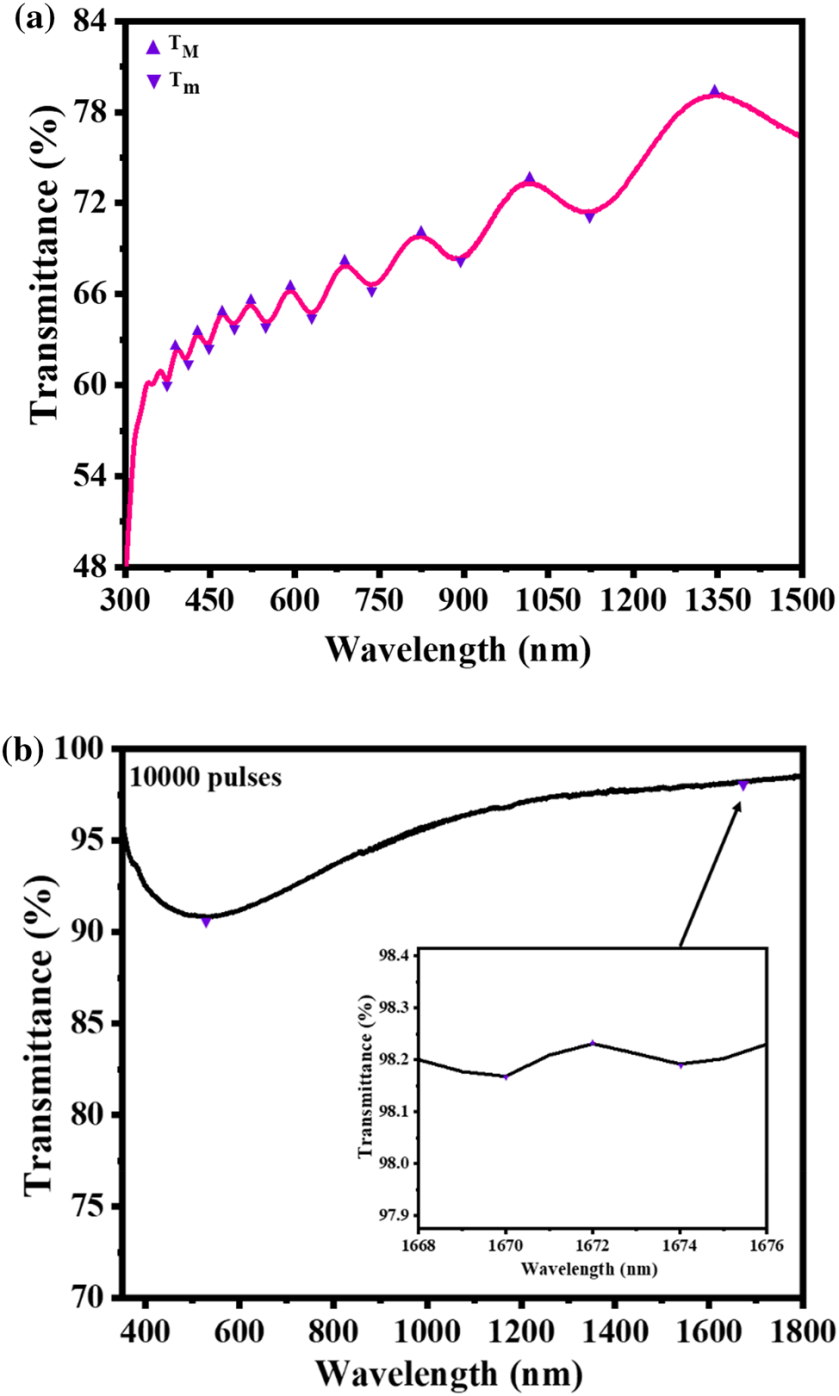
Optical transmission spectra of the film deposited at (**a**) 60,000 laser pulses and (**b**) 10,000 pulses. The top and bottom transmittance T_M_ and T_m_ are shown as filled (▲▼) triangles.

Swanepoel method^51^ was used to estimate the thicknesses of the thin films from the transmittance data. This method relies on the oscillating peak's maximum and minimum transmissions (T_M_, T_m_) as shown in Fig. 11a,b. Transmission spectra have two regions as per absorption in the films in general; region of strong absorption, and weak and medium absorption. According to Swanepoel’s method, the refractive index (n_1_) value of the thin films in the medium and weak absorption is given by^52^:

2 $${n}_{1}=[N+({N}^{2}-{S}^{2}{)}^ {{1}\left/ {2}\right.}]^{{1}\left/ {2}\right.}$$

where

3 $$N=2S\frac{{T}_{M}-{T}_{m}}{{T}_{M}{T}_{m}}+\frac{{S}^{2}+1}{2}$$

And S is the refractive index of the substrate.

Taking the basic equation of interference fringes into account:

4 $$2nd=m\lambda$$

where n is the refractive index, d is the thickness of the thin film, m is the interference order (half-integer for minima and integer for maxima) and λ is the wavelength. The thickness of the film $d$ can be estimated from the refractive indices (n_1_, n_2_) at two adjacent maxima or minima at λ_1_ and λ_2_ with λ_1_ > λ_2_ through^51^:

5 $$d=\frac{{\lambda }_{1}{\lambda }_{2}}{{\lambda }_{1}{n}_{2}-{\lambda }_{2}{n}_{1}}$$

**Figure 12:**
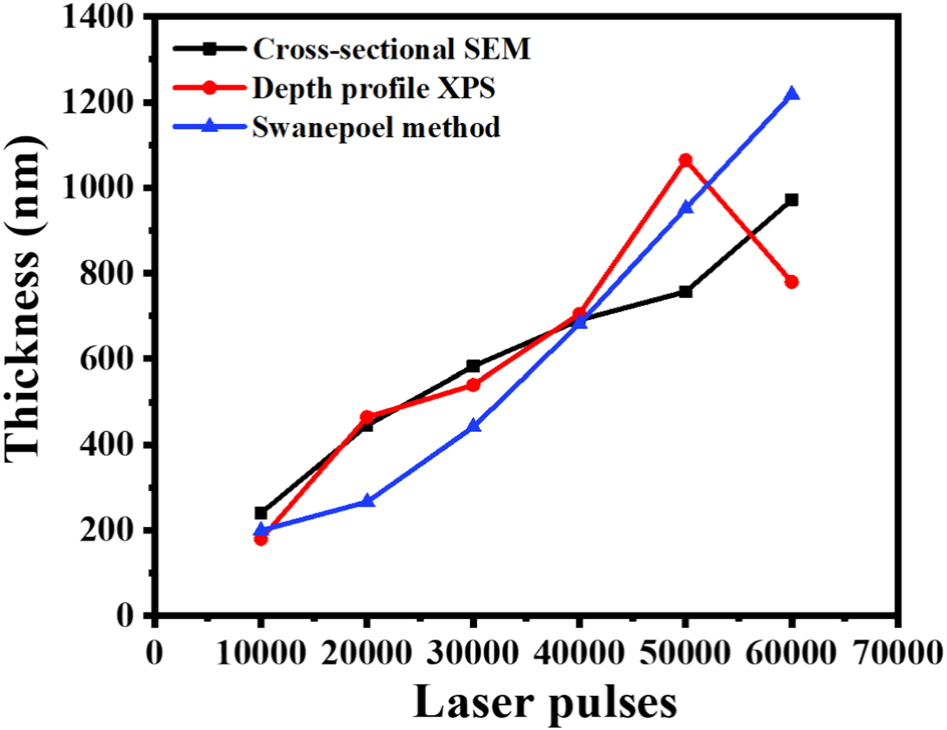
Comparison of the thicknesses as determined by SEM, XPS, and Swanepoels method.

The thickness values of the thin films estimated by the Swanepoel method were found to be 200, 267, 442, 682, 952, and 1218 nm for 10,000, 20,000, 30,000, 40,000, 50,000, and 60,000 pulses, respectively, see Table 2. It can be seen that the films became thicker as the laser pulses increased. The discrepancy observed from the Swanepoel method arose due to the interference fringes that were taken into account. Swanepoel’s approach does not always result in a good accuracy of the films’ thickness when few fringes are considered^53^, but clearly showed the same trend of increasing thickness with an increase in the number of pulses. The difference in thickness, if SEM and XPS are compared, might be due to the uneven features on the surface that can influence the thickness, especially with the XPS depth profiles. Figure 12 shows the comparison of the thicknesses as determined by SEM, XPS, and Swanepoels method. The Swanepoel method can give a rough indication of the thickness without any destructive measurements of the thin films with an underestimation at thinner films and an overestimation at thicker films.

### UC analysis

**Figure 13:**
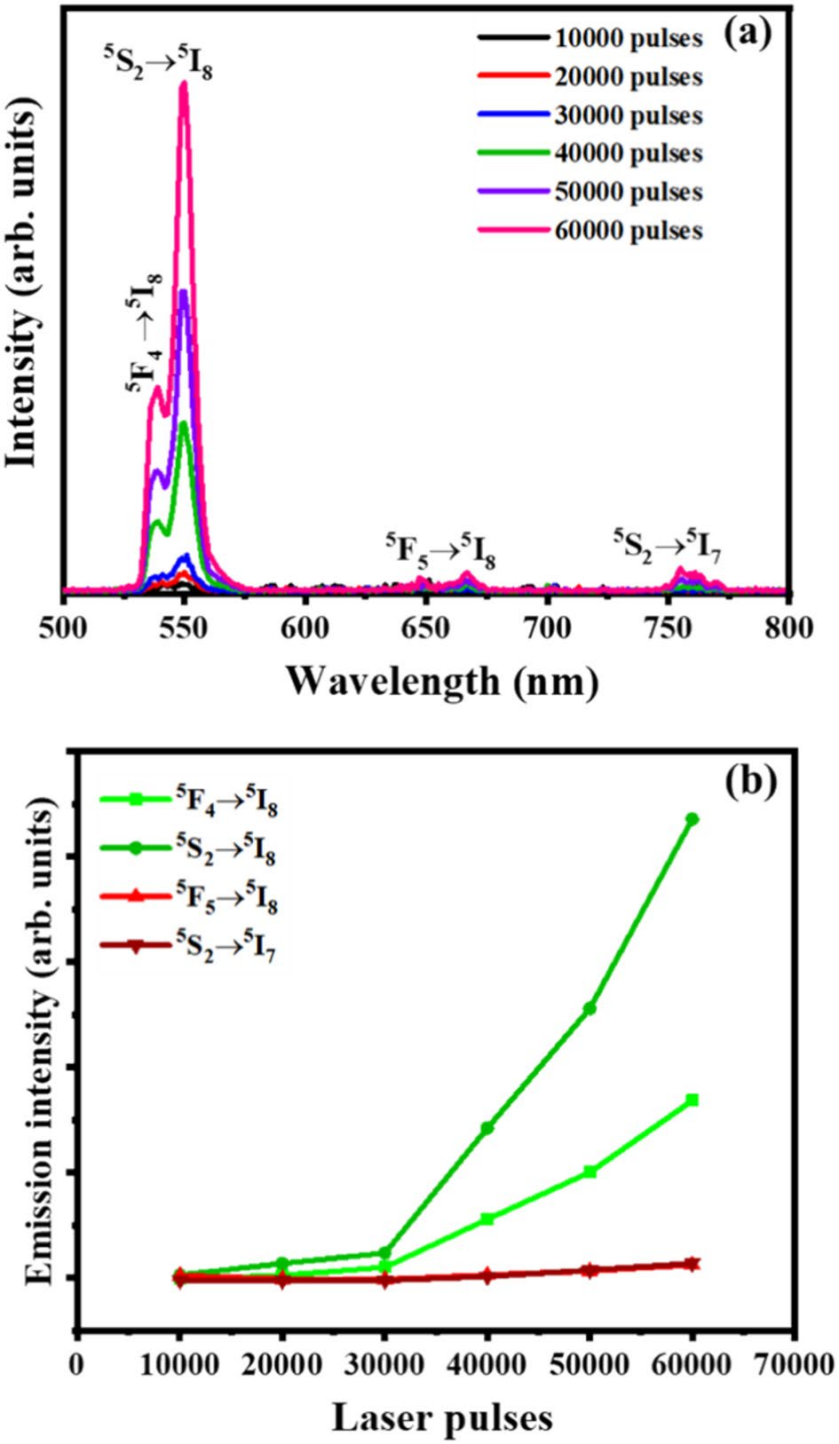
(**a**) UC emission for Y_2-x-y_O_3_:Ho_x=0.005_,Yb_y=0.05_ films, and (**b**) the UC emission intensity of Ho^3+^ ion as a function of the number of laser pulses.

The UC emission spectra of the thin films at different laser pulses under 980 nm excitation are presented in Fig. 13a. The UC emission exhibited intense green emission bands assigned to the ^5^F_4_ → ^5^I_8_ and ^5^S_2_ → ^5^I_8_ transitions at 538 and 550 nm as well as weaker red and infrared emissions at 666 and 756 nm corresponding to the ^5^F_5_ → ^5^I_8_ and ^5^S_2_ → ^5^I_7_ transitions of the Ho^3+^ ions, respectively^24^. It is observed that the UC emission intensity depends on the number of laser pulses. From Fig. 13b, it can be seen that the UC emission intensity of all emission bands increased with an increase in the number of laser pulses. The enhancement in UC emission performance is related to the reduction of internal reflections due to the surface roughness^54^. Jafer et al.^55^ observed such luminescence improvement in the Y_2_O_3_:Bi^3+^ films due to the crystallinity and the surface roughness of the prepared films. Therefore, the UC emission intensity enhancement with an increasing number of laser pulses can also be due to the crystallinity improvement of the thin films (Fig. 1a).

**Figure 14:**
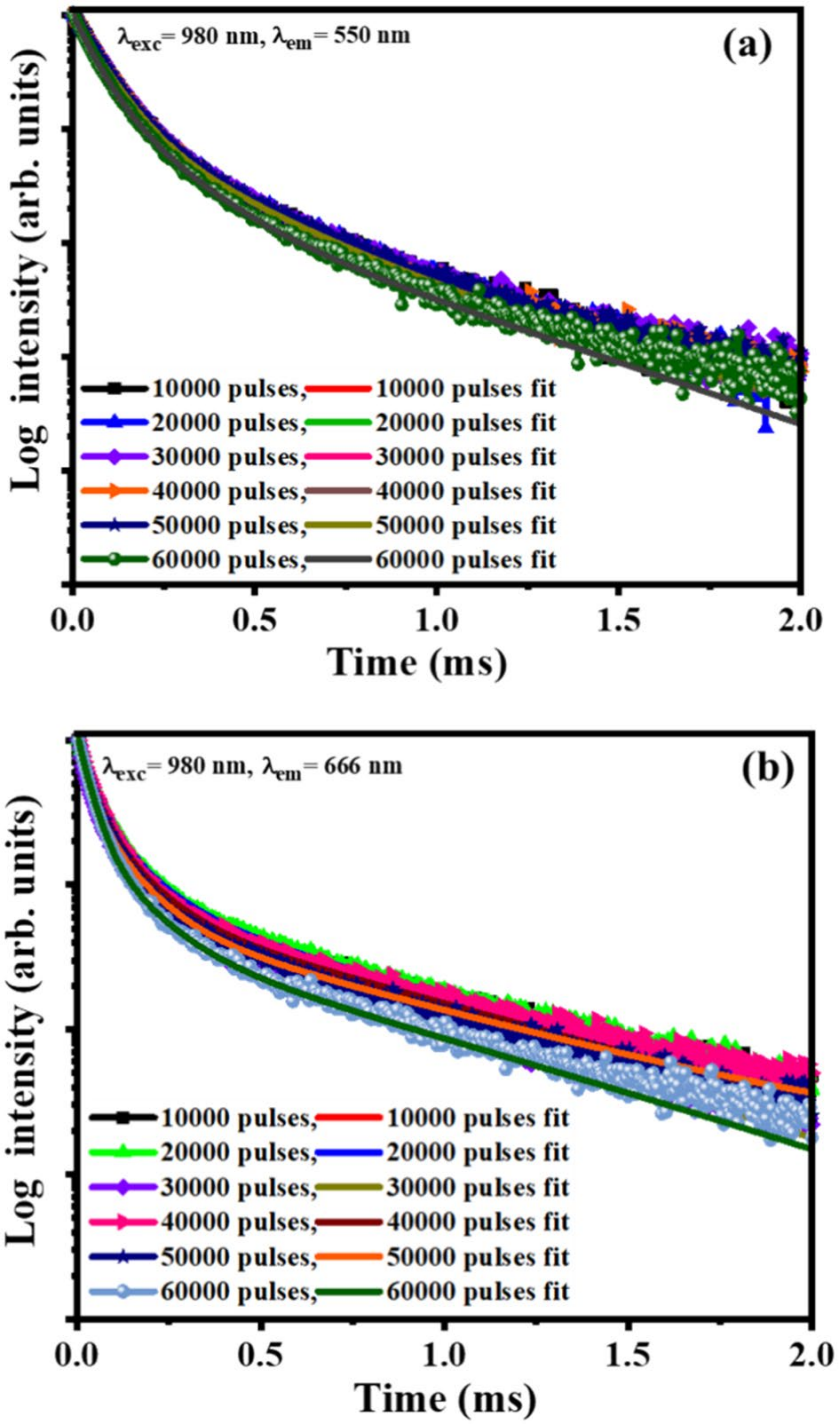
Fitted decay curves of the films monitored at (**a**) 550 nm and (**b**) 666 nm under 980 nm excitation.

The luminescence decay curves of the ^5^S_2_ → ^5^I_8_ (550 nm) and ^5^F_5_ → ^5^I_8_ (666 nm) transitions of the Ho^3+^ ion under 980 nm excitation are displayed in Fig. 14a,b.

All the luminescence decay curves were fitted with a triple exponential function^56^:

6 $$I=A\text{exp}\left(-\frac{t}{{\tau }_{1}}\right)+B\text{exp}\left(-\frac{t}{{\tau }_{2}}\right)+C\text{exp}\left(-\frac{t}{{\tau }_{3}}\right)$$

where $I$ is the intensity at time $t$, $A, B,$ and $C$ are the fitting constants, and ${\tau }_{1}, {\tau }_{2},$ and ${\tau }_{3}$ are the lifetime constants. The average lifetime (${\tau }_{ave})$ were determined using ${\tau }_{ave}=(A{{\tau }_{1}}^{2}+B{{\tau }_{2}}^{2}+C{{\tau }_{3}}^{2})/(A{\tau }_{1} +B{\tau }_{2}+C{\tau }_{3})$. The multiexponential decay curves' behavior has been reported to commonly arise from doping ions occupation in the host lattice, ion-ion interactions between the donor and the activator, and the probability of non-radiative transition between ion–ion pairs^57–59^. It can be seen that the laser pulses slightly influenced the luminescence lifetime of the films, Table 3. The lifetimes of the ^5^S_2_ → ^5^I_8_ (550 nm) and ^5^F_5_ → ^5^I_8_ (666 nm) levels decreased as the number of laser pulses increased.

**Table 3:** The lifetimes of the ^5^S_2_ → ^5^I_8_ (550 nm) and ^5^F_5_ → ^5^I_8_ (666 nm) transitions of the Ho^3+^ ion of the Y_2-x-y_O_3_:Hox_=0.005_, Yb_y=0.05_ films at different laser pulses.

Number of laser pulses	Lifetime, $\tau$ (ms)	Lifetime, ${\tau }_{ave}$ (ms)	Lifetime, $\tau$ (ms)	Lifetime,${\tau }_{ave}$ (ms)
	**550 nm emission**		**666 nm emission**	
10,000	${\tau }_{1}=0.06, {\tau }_{2}=0.24, {\tau }_{3}=0.58$	0.16	${\tau }_{1}=0.03, {\tau }_{2}=0.09, {\tau }_{3}=0.63$	0.35
20,000	${\tau }_{1}=0.07, {\tau }_{2}=0.20, {\tau }_{3}=0.50$	0.16	${\tau }_{1}=0.04, {\tau }_{2}=0.12, {\tau }_{3}=0.65$	0.34
30,000	${\tau }_{1}=0.07, {\tau }_{2}=0.22, {\tau }_{3}=0.52$	0.16	${\tau }_{1}=0.03, {\tau }_{2}=0.11, {\tau }_{3}=0.65$	0.33
40,000	${\tau }_{1}=0.07, {\tau }_{2}=0.20, {\tau }_{3}=0.48$	0.16	${\tau }_{1}=0.03, {\tau }_{2}=0.10, {\tau }_{3}=0.61$	0.30
50,000	${\tau }_{1}=0.07, {\tau }_{2}=0.19, {\tau }_{3}=0.47$	0.15	${\tau }_{1}=0.02, {\tau }_{2}=0.08, {\tau }_{3}=0.60$	0.28
60,000	${\tau }_{1}=0.05, {\tau }_{2}=0.13, {\tau }_{3}=0.41$	0.14	${\tau }_{1}=0.03, {\tau }_{2}=0.11, {\tau }_{3}=0.59$	0.25

**Figure 15:**
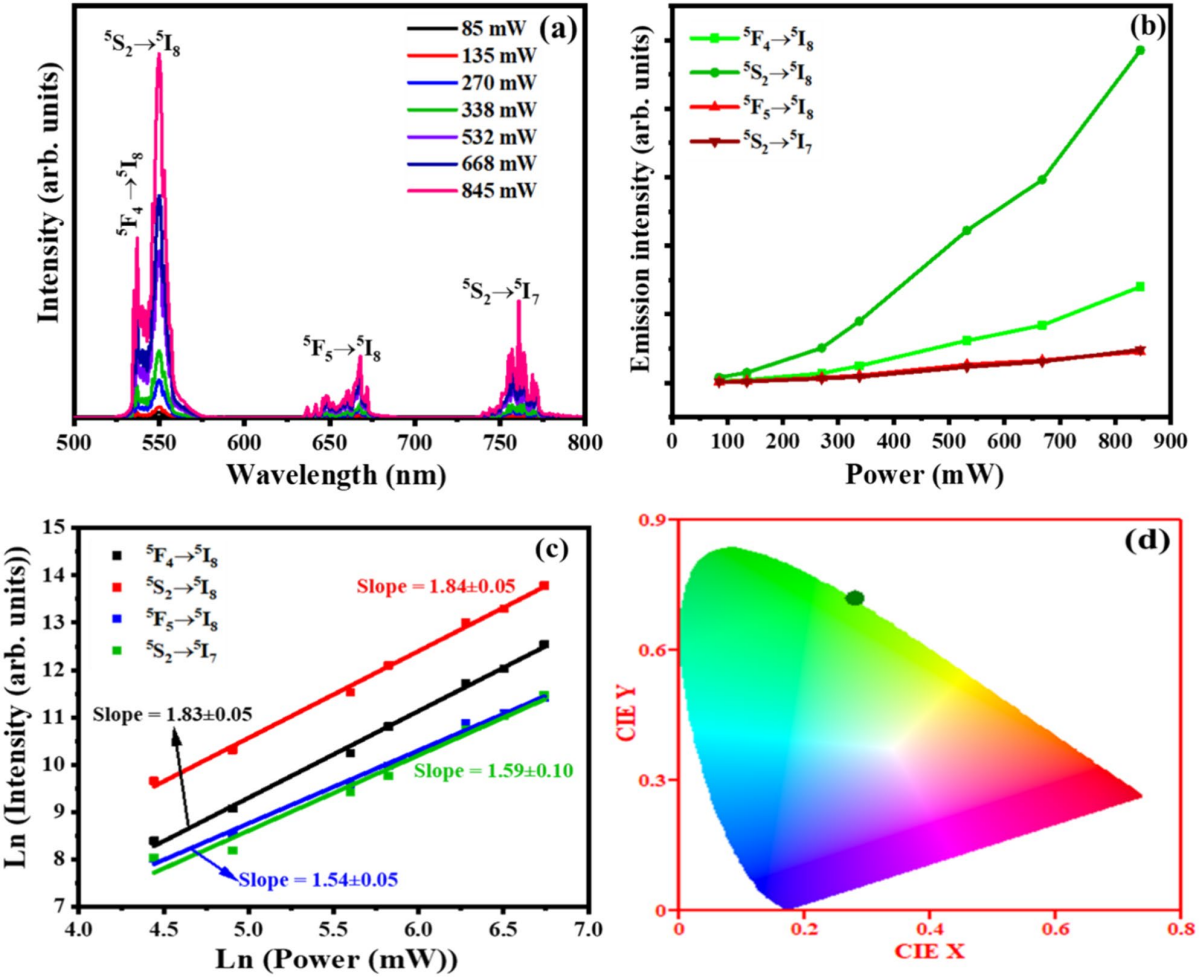
(**a**) UC emission of the films at different excitation laser power, (**b**) power dependences of the UC intensities for the green, red, and infrared bands, (**c**) Ln intensity versus Ln (Power) plot, and (**d**) CIE chromaticity coordinates of the film at 845 mW power.

**Figure 16:**
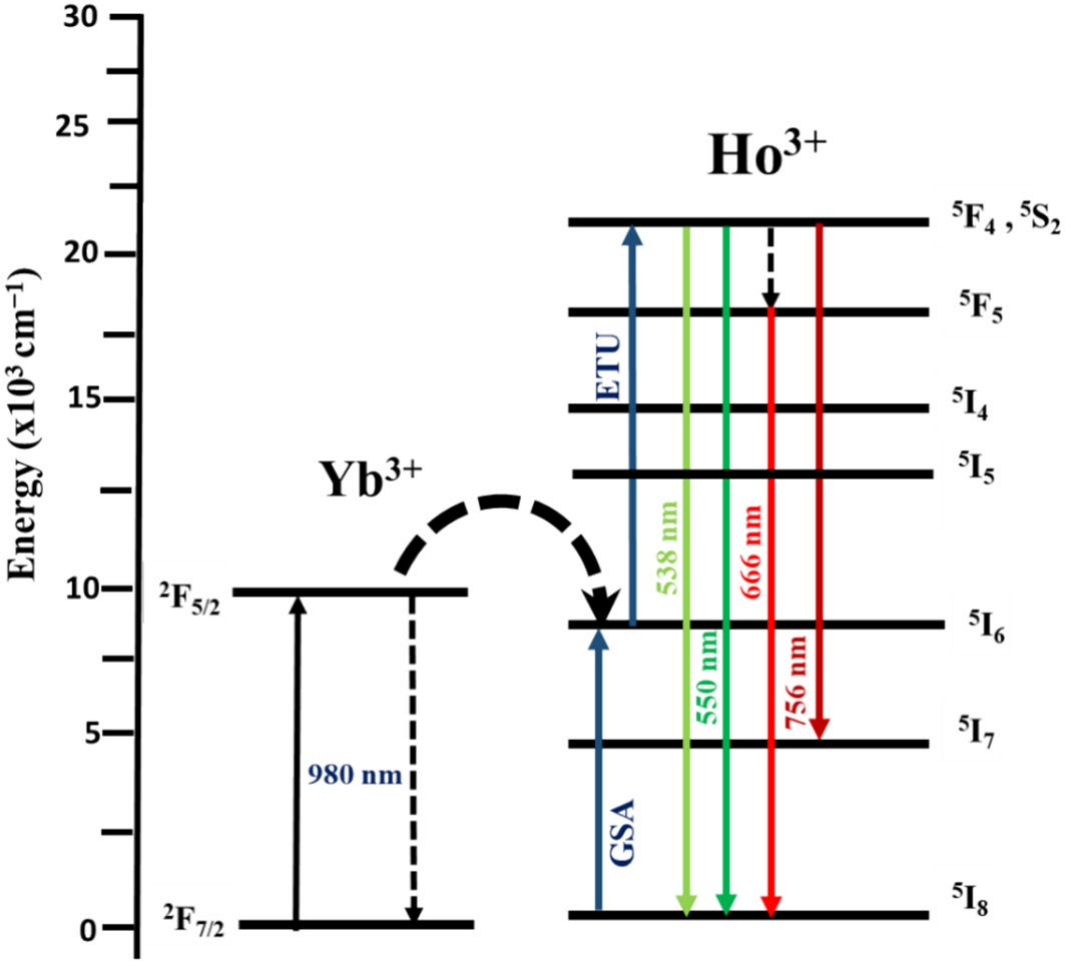
A schematic diagram of the UC mechanism in the Y_2_O_3_:Ho^3+^, Yb^3+^ thin films.

The UC emission mechanism was further studied at various excitation laser powers to determine the excited number of photons needed for each emitted photon. Figure 15a presents the UC emission spectra of Y_2-x-y_O_3_:Ho_x=0.005_,Yb_y=0.05_ film at 60,000 pulses under different pumping power of 980 nm laser. The UC emission spectra display four emission bands, a strong green (~ 538 nm), dominant green (~ 550 nm), weak red (~ 666 nm), and weak infrared (~ 756 nm) assigned to ^5^F_4_ → ^5^I_8_, ^5^S_2_ → ^5^I_8_, ^5^F_5_ → ^5^I_8_, and ^5^S_2_ → ^5^I_7_ transitions of the Ho^3+^ ions, respectively^60^. From Fig. 15b, the increase in excitation power significantly enhanced the UC emission intensity of each emission band. This UC emission enhancement is due to the increase of populations in the excited states of the dopant ions^23^. The number of excitation photons needed for each UC emission band can be determined from the relation $I \alpha {P}^{n}$^61^, where $I$ is the emission intensity, $P$ is the pumping power, and $n$ is the number of photons. In Fig. 15c, the $In \left(power\right)$–$In (intensity)$ plot has $n$ as the slope of the straight lines fit, which was found to be 1.83, 1.84, 1.54, and 1.59 for the 538, 550, 666, and 756 nm UC emission peaks. These $n$ values are close to 2 suggesting that a two-photon UC process is responsible for the observed ^5^F_4_ → ^5^I_8_, ^5^S_2_ → ^5^I_8_, ^5^F_5_ → ^5^I_8_, and ^5^S_2_ → ^5^I_7_ emissions. Figure 15d shows the Commission International de I’Eclairage (CIE) chromaticity diagram of the film under 980 nm laser excitation at 845 mW power. The calculated CIE coordinates (0.30, 0.71), estimated using UC emission spectra (Fig. 15a), shows that the UC emission has a pure green colour. The UC luminescence of Y_2-x-y_O_3_:Ho_x=0.005_,Yb_y=0.05_ occurred following the UC mechanism shown in Fig. 16. In this process, the Yb^3+^ ion is excited from the ^2^F_7/2_ ground state via the 980 nm excitation to the ^2^F_5/2_ excited state. Due to the close energy match between ^2^F_5/2_ (Yb^3+^) and ^5^I_6_ (Ho^3+^), the excited Yb^3+^ ion can easily transfer the absorbed energy to the ^5^I_6_ excited level of Ho^3+^ ion via non-radiative resonance energy transfer. Energy transfer upconversion (ETU) further excites the electron in the ^5^I_6_ level to the ^5^F_4_, ^5^S_2_ levels of the Ho^3+^, which results in intense green emission through ^5^F_4_ → ^5^I_8_ and ^5^S_2_ → ^5^I_8_ transitions at 538 and 550 nm, and weaker infrared emission through the ^5^S_2_ → ^5^I_7_ transitions at 756 nm. Furthermore, weak red emission at 667 nm through ^5^F_5_ → ^5^I_8_ transitions was observed after the ^5^F_5_ level may have been populated by the non-radiative multiphonon relaxation process from the ^5^F_4_, ^5^S_2_ levels^62^.

## Conclusions

High-quality Y_2-x-y_O_3_:Ho_x=0.005_,Yb_y=0.05_ thin films have been deposited on soda-lime glass substrates at various numbers of laser pulses using the PLD technique. It was discovered that the number of laser pulses in the films has a significant impact on crystallinity, surface properties, and UC luminescence. The XRPD results showed the films were formed as a single-phase cubic structure of Y_2_O_3_ with different preferred plane orientations under various laser pulses. High-resolution XPS confirmed the two sites of Y_2_O_3_. The thin film thicknesses were determined by cross-sectional SEM measurements, depth profile XPS, and the Swanepoel method. The cross-sectional SEM thicknesses were determined to be 241, 446, 583, 691, 757, and 972 nm for 10,000, 20,000, 30,000, 40,000, 50,000, and 60,000 pulses, respectively. The depth profile XPS thicknesses were determined to be 180, 465, 540, 705, 1065, and 780 nm for 10,000, 20,000, 30,000, 40,000, 50,000, and 60,000 pulses, respectively. The Swanepoel method thicknesses were determined to be 267, 442, 682, 952, and 1218 nm for 20,000, 30,000, 40,000, 50,000, and 60,000 pulses, respectively.

The thicknesses of the films determined by cross-sectional SEM, depth profile XPS, and the Swanepoel methods indicated that the films became thicker as the laser pulses increased. Power dependence and lifetime measurements confirmed the involvement of the two-photon UC process. The UC luminescence showed a dominant green emission under 980 nm excitation, making the Y_2-x-y_O_3_:Ho_x=0.005_,Yb_y=0.05_ UC transparent thin films phosphor a good candidate for solar cell applications.

## Data Availability

Data is not publicly available, but data will be made available on request.
